# Supplementary material for: Childhood sexual abuse, alcohol and drug use problems among Black sexual minority men in six US Cities: Findings from the HPTN 061 study
Source: PLoS One. 2022 Dec 22;17(12):e0279238. doi: 10.1371/journal.pone.0279238 (PMC9778556; doi:10.1371/journal.pone.0279238)
Supplement: Attachment — Submitted filename: Response to reviewer comments submitted.docx [file pone.0279238.s001.docx]

Thank you for this opportunity to resubmit this manuscript to PLOS ONE. Below we respond to each of the comments provided by the reviewer and academic editor. We have bolded all the revisions in the revised manuscript.

**Academic editor**

We note that the grant information you provided in the ‘Funding Information’ and ‘Financial Disclosure’ sections do not match. When you resubmit, please ensure that you provide the correct grant numbers for the awards you received for your study in the ‘Funding Information’ section

**Response**: **The funding institutes that supported the HIV Prevention Trials Network (HPTN) are National Institute of Allergy and Infectious Diseases (NIAID), National Institutes of Child Health and Human Development (NICH/HD), National Institute of Mental Health (NIMH), Office of AIDS Research, National Institutes of Health (NIH), Department of Health and Human Services under cooperative agreement UM1- AI068619, UM1-AI068617, and UM1- AI068613.**

**The funders had no role in study design, data collection and analysis, or preparation of the manuscript.**

Please remove any funding-related text from the manuscript and let us know how you would like to update your Funding Statement. Currently, your Funding Statement reads as follows:

“HPTN 061 grant support was provided by the National Institute of Allergy and Infectious Disease (NIAID), National Institute on Drug Abuse (NIDA) and National Institute of Mental Health (NIMH): Cooperative Agreements UM1 AI068619, UM1 AI068617, and UM1 AI068613.  Additional site funding –Fenway Institute CRS: Harvard University CFAR (P30 AI060354) and CTU for HIV Prevention and Microbicide Research (UM1 AI069480); George Washington University CRS: District of Columbia Developmental CFAR (P30 AI087714); Harlem Prevention Center CRS and NY Blood Center/Union Square CRS: Columbia University CTU (5U01 AI069466) and ARRA funding (3U01 AI069466-03S1); Hope Clinic of the Emory Vaccine Center CRS and The Ponce de Leon Center CRS: Emory University HIV/AIDS CTU (5U01 AI069418), CFAR (P30 AI050409) and CTSA (UL1 RR025008); San Francisco Vaccine and Prevention CRS: ARRA funding (3U01 AI069496-03S1, 3U01 AI069496-03S2); UCLA Vine Street CRS: UCLA Department of Medicine, Division of Infectious Diseases CTU (U01 AI069424. The funders had no role in study design, data collection and analysis, decision to publish, or preparation of the manuscript.”

**Response: Thank you for this comment. As recommended, we have removed the funding information from the manuscript.**

Please include your amended statements within your cover letter; we will change the online submission form on your behalf.

**Response: this is the amended statement of funding information “The funding institutes that supported the HIV Prevention Trials Network (HPTN) are National Institute of Allergy and Infectious Diseases (NIAID), National Institutes of Child Health and Human Development (NICH/HD), National Institute of Mental Health (NIMH), Office of AIDS Research, National Institutes of Health (NIH), Department of Health and Human Services under cooperative agreement UM1- AI068619, UM1-AI068617, and UM1- AI068613.**

5.In your Data Availability statement, you have not specified where the minimal data set underlying the results described in your manuscript can be found. PLOS defines a study's minimal data set as the underlying data used to reach the conclusions drawn in the manuscript and any additional data required to replicate the reported study findings in their entirety. All PLOS journals require that the minimal data set be made fully available. For more information about our data policy, please see <http://journals.plos.org/plosone/s/data-availability>.

**Response: HPTN have procedures in place, here is the the request**

Upon re-submitting your revised manuscript, please upload your study’s minimal underlying data set as either Supporting Information files or to a stable, public repository and include the relevant URLs, DOIs, or accession numbers within your revised cover letter. For a list of acceptable repositories, please see <http://journals.plos.org/plosone/s/data-availability#loc-recommended-repositories>. Any potentially identifying patient information must be fully anonymized.

Important: If there are ethical or legal restrictions to sharing your data publicly, please explain these restrictions in detail. Please see our guidelines for more information on what we consider unacceptable restrictions to publicly sharing data: <http://journals.plos.org/plosone/s/data-availability#loc-unacceptable-data-access-restrictions>. Note that it is not acceptable for the authors to be the sole named individuals responsible for ensuring data access.

We will update your Data Availability statement to reflect the information you provide in your cover letter.

**Response: The data that was used for this study are available upon request from the HIV Prevention Trials Network (**[**https://www.hptn.org/research/studies/hptn061/accesstostudydata**](https://www.hptn.org/research/studies/hptn061/accesstostudydata)**).There are restrictions to the data and prevent the authors from sharing the de-identified datasets or placing them in a public database.**

Please review your reference list to ensure that it is complete and correct. If you have cited papers that have been retracted, please include the rationale for doing so in the manuscript text, or remove these references and replace them with relevant current references. Any changes to the reference list should be mentioned in the rebuttal letter that accompanies your revised manuscript. If you need to cite a retracted article, indicate the article’s retracted status in the References list and also include a citation and full reference for the retraction notice.

**Response: We have reviewed the references and updated them accordingly**

**Reviewer**

1. In general, I recommend using "sexual minority men" (and the abbreviation SMM) rather than "men who have sex with men" throughout. The former is more directly applicable in the context of minority stress theory, as it's more identity centered (and still includes MSM). Note that it's fine to use MSM when referring to a previous study that used that specific term, but otherwise I strongly recommend using SMM.

**Response: As suggested, we have reviewed the manuscript and replaced the term “MSM” with sexual minority men (SMM).**

2. I would explicitly state that the higher risk of alcohol and drug use is related to social and structural stressors in the first sentence, as otherwise it's easy for a reader to skim the first few lines and misunderstand this (even though you otherwise explain this well in paragraph 2).

**Response: We agree with the reviewer, we have clarified this in the revised introduction. We have included “The reasons for the disproportionate risks to these conditions among BSMM are due intersecting structural adversities, including social stressors”**

3. I recommend adding the OR and confidence intervals for income and rent towards the end of the last results paragraph, as it looks like you added them for all the other variables.

**Response: Thank you for pointing this out. We have added odds ratio and confidence intervals for income and rent in the revised results section.**

4. Minor point: You may want to use BSMM when discussing Black sexual minority men, as the acronym will come up several times throughout the paper.

**Response: Thank you for this suggestion, the revised manuscript to reflect this suggestion.**

5. A bit more on policy recommendations, particularly for Black SMM health equity efforts, is recommended.

**Response: We appreciate this suggestion. We have included this recommendation in the revised discussion section. The revised section now includes need to focus on interventions that address social and structural factors that exacerbate health inequities in vulnerable and stigmatized populations like BSMM**

Similarly, a bit more of future research directions is recommended.

**Response: Yes, we have added more details on areas for future research direction in the discussion section. We have added the need for more studies to examine the role of intersecting social determinants of health and health outcomes of vulnerable and stigmatized populations like BSMM.**

6. Minor point: Reference font should be changed to match the main text.

   **Response: Thank you for noting this, we have corrected the reference font to align with the main text.**
